# Commercialization of biopharmaceutical knowledge in Iran; challenges and solutions

**DOI:** 10.1186/2008-2231-22-29

**Published:** 2014-02-24

**Authors:** Nasser Nassiri-Koopaei, Reza Majdzadeh, Abbas Kebriaeezadeh, Arash Rashidian, Mojtaba Tabatabai Yazdi, Saharnaz Nedjat, Shekoufeh Nikfar

**Affiliations:** 1 Department of Pharmacoeconomics and Pharmaceutical Administration, Faculty of Pharmacy, Tehran University of Medical Sciences, Tehran, Iran; 2 Knowledge Utilization Research Centre and School of Public Health, Tehran University of Medical Sciences, Tehran, Iran; 3 Pharmaceutical Policy Research Center, Faculty of Pharmacy, Tehran University of Medical Sciences, Enqelab Square, Tehran, Iran; 4 Knowledge Utilization Research Centre and Department of Health Management and Economics, School of Public Health, Tehran University of Medical Sciences, Tehran, Iran; 5 Department of Pharmaceutical Biotechnology, Faculty of Pharmacy, Tehran University of Medical Sciences, Tehran, Iran

**Keywords:** Knowledge translation, Biopharmaceutical research, Facilitators and barriers

## Abstract

**Background:**

The objective of this study was to investigate the application of the university research findings or commercialization of the biopharmaceutical knowledge in Iran and determine the challenges and propose some solutions.

**Results:**

A qualitative study including 19 in-depth interviews with experts was performed in 2011 and early 2012. National Innovation System (NIS) model was employed as the study design. Thematic method was applied for the analysis. The results demonstrate that policy making, regulations and management development are considered as fundamental reasons for current commercialization practice pattern. It is suggested to establish foundation for higher level documents that would involve relating bodies and provide them operational guidelines for the implementation of commercialization incentives.

**Conclusions:**

Policy, regulations and management as the most influential issue should be considered for successful commercialization. The present study, for the first time, attempts to disclose the importance of evidence input for measures in order to facilitate the commercialization process by the authorities in Iran. Overall, the NIS model should be considered and utilized as one of the effective solutions for commercialization.

## Introduction

Biotechnology science has proven to be a fast growing source of new technologies and innovative medicines for the pharmaceutical industries during the recent decades [1]. Biopharmaceuticals is presenting itself as the future promise for the safer, targeted and curative medicines opening the scope for treatment of rare and incurable health conditions [2]. New pharmaceutical companies are emerging with a portfolio of biotechnology drug candidates, while the current market leaders are transforming their pipelines to biotechnology derived lead compounds. The specific feature of biotechnology, in general, in being based largely on academic and university researches renders the commercialization of research finding substantial importance [3]. The sufficient research budgets and innovation commercialization expertise to remains as an ever growing issue to be addressed [4]. Speculating the Iranian Pharmaceutical market, which has been substantially growing during past two decades, demonstrates that medical expenditures has increased as part of overall health system costs [5-7]. There are a satisfactory number of research institutes in the biopharmaceutical field in Iran, but the number of biopharmaceutical companies has been limited to few ones. One possible explanation for the perceived gap between research and production might be the malfunction of the commercialization practice. In the fields, including biopharmaceuticals, where an essential part of knowledge creation takes place in universities and research institutes, the fruitful application of the knowledge needs a transformation process, which involves inventing uses for new scientific ideas and making products or services possible out of them [8,9]. Many universities have persuaded their academic staff and researchers to commercialize their findings and have established university-industry cooperation offices to potentiate these activities, but these mere initiatives are not adequate tools for assuring commercialization prosperity [10]. Very few research finding have been successful enough to turn into commercialized products [4]. Moreover different goals and terminology wielded in academic and industrial contexts, a function of translation to render the two sides a dual comprehension capability is required. So prior to a simple transfer of knowledge, we need an active mechanism making the newly formed knowledge understandable and available for the various technical contexts [11]. Biopharmaceutical industry, among the other high tech ones, encounters with the growing and uncertainty inherent in technological changes, difficulties in handling forthcoming changes due to the immaturity of the industry and the shortage of intrinsic management skills. In addition, dependent on the well-established pharmaceutical and chemical industries, biopharmaceutical industry needs to be assisted with introducing its developing products into market place [12]. All these issues render knowledge translation a substantial importance. In Iran, biopharmaceutical researches have a remarkable history; from when Pasteur and Razi institutes were established in 1919 and 1930, respectively [13]. Recently, more research institutes and academic groups have been engaged in biopharmaceutical research. Some biopharmaceutical corporations have been founded in early previous decade. The number has grown but some experts believe that this capacity can be further potentiated. University-industry relationship has been a topic of concern in Iran. To date some steps have been taken to strengthen such a relation, but outcomes might not fulfill the primary goals. In the present study we aim at investigating the commercialization of the biopharmaceutical knowledge in Iran and determining the barriers and then proposing facilitations possible to further strengthen university knowledge utilization.

## Methods

The present qualitative study, in 2011 and early 2012, was carried out on some groups: biopharmaceutical professionals, policy makers and some activists involved in the industry, a purposive sampling method was admitted and included 19 in-depth interviews. Ethics Committee approval was received for the study from Tehran University of Medical Sciences research deputy. Involvement in research and development, science and technology or drug policy making level, industrial and business background were considered while defining the subgroups of the study. The study’s subgroups are shown in Table 1. Researchers were selected from the faculty members of Tehran University of Medical Sciences and Pasteur institute of Iran. Both the academic organizations possess biopharmaceutical department, conducting researches and offer PhD degrees in pharmaceutical biotechnology. These instruction and research organizations have great background in the biopharmaceuticals in Iran and the most important biopharmaceutical companies in Iran have derived from these institutes. In-depth interviews were held with all the participants in the study. Interviews and discussions on the questions listed in Table 2 were done until the point of saturation. Totally 19 individual in-depth interviews, each lasting for 0.5 to 1.5 h, were conducted. Data gathering on the research output of the research organizations were done, regarding the outcome of the current status of putting research into action in two important research institutes. In this study, term definition was as follows: linking research to action or knowledge translation has been considered as the collection of activities that range from ‘designing the research question up to application of its results with the aim of improving health and healthcare services [14] and technology commercialization is the process of bringing technical innovation to the marketplace. In the study, the barriers and solutions of knowledge translation in the biopharmaceutical sector have also been studied. Due to the active role of the researcher as a researcher at the department of pharmaceutical biotechnology at the faculty of pharmacy, Tehran University of Medical Sciences, the interviewer became a research fellow observer for the Tehran University of Medical Sciences as a typical example of Iranian research institutes being analyzed in this study [15]. Some interviewees function simultaneously or have background in different positions such as academic staff, industry shareholder and manager or policy making that causes the lack of separation in the sample through different groups.

**Table 1:** The groups interviewed in the study on ‘Commercialization of biopharmaceutical knowledge in Iran’

**Group**	**Subgroup’s characteristics**	**Individuals interviewed**
Researcher and academics	Researchers in units under MOHME’s authority	Academic staff and researchers from Tehran University of Medical Sciences, Pasteur institute, Tehran university, Shahed university
	Researchers in units under authority of Ministry of science and technology	
Policy makers	Pharmaceutical market managers and policy makers in FDO and related organizations	MOHME’s Deputy of Research and Technology, Ex-director general of deputy of Food and Drug, Office of health technology at MOHME, Office of research development at MOHME, Office of biological products at FDO, Feasibility study manager at the Ministry of Industry, mine and commerce, Governmental Insurance company manager, Chancellor of faculty of pharmacy, Biopharmaceutical production unit managers, industrial factories
	Research managers and policy makers in MOHME and related organizations	
Biopharmaceutical Industry experts	Experts in the biopharmaceutical industry	

MOHME, Ministry of Health and Medical Education.

**Table 2:** Questions to conduct the in dept interviews

	**Question**
1	What is your perspective on commercializing university research findings in the field of biopharmaceutical knowledge in Iran?
2	What are the main challenges in commercialization of university research findings in the field of biopharmaceutical knowledge in Iran? How do you assess them?
3	What do you propose to meet these challenges? And what will be the ideal situation of biopharmaceutical knowledge commercialization in Iran?

### Data analysis

The in-depth interviews were documented by a note taker and audio-recorded. Qualitative analysis was performed through the thematic method; the documented interviews and their transcripts were studied several times by the interviewer. Thereafter thematic coding process was done on the interviews and their transcripts by the interviewer while other members of the team regarded the themes and the categories were extracted [15].

## Results

The eight categories extracted in the study were policy making, regulations and management development, investment and financial contribution, improving the research capability, human resource development, encouraging the entrepreneurship, industrial manufacturing capacity, promoting the social culture and values and extending the relations (Table 3). These categories were extracted while the National Innovation System model was admitted as the cornerstone for the structure of thematic analyses and formulation of categories. In each sector the challenges and barriers are presented and then the facilitations possible.

**Table 3:** Categories and codes extracted from the in dept interviews

**No.**	**Category**	**Code**
1	Policy making, regulations and management development	Policy making, legislation and management procedure reform
		Reform in authorities
		Transparency
		Concentration in policy making and legislation
		Stability
		Management challenges
		Market control and expansion policies
		Standards setting
		Control and auditing regulations
		Facilitating regulations
		Prioritization
2	Investment and financial contribution	Financial support management and targeting
		Private sector capacity use
		Private investment
3	Improving the research capability	Purposeful research
		Research and education system reform
4	Extending the relations	Scientific, technological and trade relations
		Interaction between the authorities and consistent approach
		International relations
5	Human resource development	Instruction and evaluation
		Provocation and leading
		HR pyramid development
6	Encouraging the entrepreneurship	Entrepreneurial training and culture
		Commercialization and entrepreneurial facilitation
		Professional commercialization and entrepreneurial services
7	Industrial manufacturing capacity	Knowledge and corporation management
		Pharmaceutical industry renewal and amendment
8	Promoting values (attitudes) and the public culture	Providence
		Cooperation, perseverance and lack of exaggeration
		National production support
		Innovation and risk taking

### Policy making, regulations and management development

#### Challenges

According to the topics noticed by the interviewees, the most critical factors affecting the commercialization of the biotechnology in Iran lie within this category, as expressed by all. One of the academic staff said:

“We have everything but one; that is management!”

In the past twenty years, a great amount of policies have been made to promote biotechnology commercialization but there has been no organized structure to monitor and document their outcomes. Short term decisions to react to day-by-day issues have been prevalent. Prioritization in the national needs has been lacking. Nearly all experts agreed that management practice and method has slowed down the process by obscure protocols, extensive bureaucracy, outdated technology, lacking predefined and clear workflow and very long period to acquire new knowledge impeded smooth progress. Moreover, standards and criteria for performance were not well defined or lacking. Planning and defining targets did not comply with scientific principles of management and in most cases based on personal preferences. One manager at a science park said that:

“Incubators are managed through personal preferences without any performance criteria.”

Legislation has not followed specific methodology and practice. Most interviewees emphasized that:

“Introduction of intellectual property law should be regarded as a basement factor.”

Conflicting regulations, very late legislation, ambiguous and interpretable regulations and regulations opposing the overall national laws and policies were among the most vital issues noted. On the other hand the authorities in charge of the pharmaceutical sector engaged themselves in the medicine supply rather than control and auditing, that could endanger serious quality improvement by abating the competition in the market. Transparency was stressed as the underpinning reason for a substantial portion of secrecy among researchers, malfunction of the granting system and conflict of interest in the whole pharmaceutical sector. Moreover lack of assurance to implementation of regulations in practice vigorously damaged public trust, in spite of decentralized decision making, especially in granting. Regarding the unorganized manner under which supports are offered, conflicting decisions in distinct institutions, conflict of interest and consecutive management changes, dispensed policy making renders the entrepreneurial context unstable and undesirable for investment. Some interviewees believe that strict market control, pricing policy, generic medicine scheme and confinement deteriorates market power of companies. In this regard one policy maker said:

“The government of Iran had better withdraw itself from medicine supply.”

Definition of standards attracts great attention in the experts as management practices are not well controlled and the outcomes do not serve any feedback for any corrective actions. Some criteria for the scientific board promotion, as explained earlier, appear to create obstacles for innovative researches.

#### Solutions

Introduction of responsible management into the pharmaceutical sector without conflict of interest and in a stable situation was suggested. Using the course of action adopted by pioneering countries while accommodating to the local circumstances and taking advantage of domestic or international consultants can be suitable. Successes and losses pursuit and causation would be a solution. One of the industrial activists said:

"No one knows where others have gone; we progress by try and error."

Determining the national priorities and broadcasting them for all the researchers was proposed. Intellectual property right regulations were regarded as a substantial need for the productive researches, since protected rights, as the driver for efforts to do innovative researches, warrants the ultimate possible profits. Stability in the management and enforcement of the regulations and policies should be assured by the government. Briefly, management practices, regulation and policy making must be revised.

### Investment and financial contribution

#### Challenges

Financial support plays an undeniable role in conduction of purposeful research. Nevertheless, noted by most interviewees, the current flow of monetary support would not suffice further growth and assist the researches through to commercialization, except that more resources in a consistent manner be underway. Terms and conditions under which the supports are allocated is a point of controversy since there are not any transparent and predetermined procedure approved for the granting and some conflicts of interest, of course, exist. An academic staff said:

"Research budget undergoes great lobbies and suffers from conflict of interest and personal tastes."

Venture capitalist funds are neither present nor functioning properly in Iran. Almost all interviewees emphasized the supportive and facilitating role of such funds. Publishing articles has turned to the pervasive outcome of most the researches, a fact that has been criticized by a great number of research and development experts. Patenting as an alternative would better be introduced to the Iranian scientific society, especially biopharmaceuticals and other high tech fields to help them protect their research findings for probable future commercialization which needs financial contribution. Another issue, as specified by nearly all interviewee groups, which could open the way up to the commercialization and knowledge translation, is privatization in the biopharmaceutical sector. Industrialists agreed that the few presently functional corporations are private and almost all entrepreneurs prefer establishing private ones to prevent tackling the governmental bureaucratic management style. Efforts should focus on capital marketing within the private sector to attract more investment to the newly formed science based companies. One of the industrialists said:

"There is a great deal of money nested in the private sector seeking the opportunity to enter the field."

#### Solutions

So revising the resource allocation policies and earmarking them to more efficient universities and institutes and simultaneously to research priorities appointed by a joint collaboration between researchers and industrialists are strictly recommended. Financial support from patenting remains to be considered. A growing amount of grants are being awarded to the researchers especially in larger universities. One of the academic staff said:

"The government should direct its money correctly to the most efficient parts."

### Improving the research capability

#### Challenges

Increasing the number of researches and improving its content was regarded as necessary that are both applicable and purposeful in nature. Such types of researches need good research planning by the institution management. Those researches should include all fields relating to the biopharmaceutical sciences such as cellular and molecular biology, microbiology, genetic engineering and process engineering that involve facilitated communications between those disciplines. Basic sciences constitute a pivotal part of the knowledge needed in the biopharmaceutical industry which is somehow ignored. Prioritization in the biopharmaceutical sector according to the domestic market needs would aid the better research topic and field selection. In spite of the speedy movement of the universities in the world towards knowledge generation and turning that knowledge to wealth, Iranian universities are struggling with the ambiguity of role and mission and have not redirected their work flow toward that aim yet. One researcher said:

"Our universities wonder how to act; human resource production or research or commercialization."

Assuming the structural and management practice modification, universities can consolidate dynamism and dexterity in electing to research novel and high tech disciplines. Most interviewees complained about the low amount of research budget. A pharmaceutical biotechnologist added:

"With the current funds we are going nowhere!"

#### Solutions

Most universities in Iran are pursuing the human resource training goals and recently have fortunately paid attention to the research. In this regard, universities must redefine their mission and vision. Redefining and redirecting the research budgets should be considered. Searching for new financial sources especially from the private sector was deemed as a feasible solution. A technology policy maker holds that:

"We can let the private sector to establish its research institutes and substitute the governmental bodies.

### Human resource (HR) development

#### Challenges

Most interviewees emphasized that both the quantity and quality of the graduates should be regarded; proposing a national or sector-wide qualification system. Although some level of disagreement was observed in the interviewees on the qualifications of the HR, some regulators and policy makers stated that the present graduates are well trained and qualified but some others, most of whom rooted in academia, expressed that qualified and experienced graduates are few in quantity in comparison to the needs. One of the interviewees engaged in policy making believed that:

"There is quite enough number of biotechnology experts in Iran and they should be appropriately assigned and recruited."

While another interviewee as an academic staff, held that:

"We have not adequate quantity and quality workers and experts in the biotechnology industry, albeit a growing number of higher education students graduate every year."

There was remarkable agreement on the following issues, although some interviewees did not express if they were for or against. Pharmacy curriculum revision seems necessary, incorporating the fundamentals of pharmaceutical biotechnology as well as the industrial and downstream processing aspects and the fields mentioned earlier. Education system reform should also introduce more applied and experimental training rather than pure ones, a matter that is regarded as a deleterious problem with the trainings offered in the universities. Designing and establishing auxiliary courses to complement the content of pharmacy training with emphasis on the industrial and entrepreneurial aspects are necessary. Researcher^’^s welfare is another factor that can affect the proper research performance in the universities.

#### Solutions

Training more and more number of students to conduct research on the biopharmaceutical issues is an important need, a prevalent thought among the interviewees. Guiding the students for their career is a must. Applied and purposeful research is denoted as one of the most influential factors on the commercialization in Iran. One academic staff said:

"Our students amaze in their career and most of them have no plan to how to select their research field."

Leading the researchers toward the national priorities may be regarded as remarkable choice for redirecting the research. Providing incentives for applied researches as performed currently by the Iranian Nanotechnology Initiative Council (INIC), is deemed to be efficient a way.

A researcher on the academic policies said:

"Continuous training and knowledge improvement by the researchers and the industrial work fellows including managers and research and development (R&D) staff is some task that could be accomplished by the universities, as well as involvement in the technology transfer process."

### Encouraging the entrepreneurship

#### Challenges

Learning commercial thinking seems scarce and sporadic among the scientific society in Iran that may derive from the social attitude toward wealth. One policy maker as well as an academic staff said:

"In general the rich are regarded as negative and pursuing such morale equates accepting levels of stigmatization."

On the other hand some regulations had previously restricted researchers from establishment of science based companies, but recently revoked. Offering the courses and trainings relevant to entrepreneurship presently is lacking in the pharmacy and other disciplines education curriculum, even in primary and elementary schools. Designing and facilitating the procedures for commercialization was stated by nearly all the interviewees to be of substantial criticality. Presently there are no defined structures or organizations responsible for fostering the commercialization process and researchers have to personally seek some supports. One biotechnologist stated that:

"There are no paved ways to commercialization; the way is so rough that withdrawal is by far wiser."

The role of the existing incubators and science and technology parks are somehow ambiguous and undefined. These entrepreneurial centers function less efficiently however successful progenitors do exist. Providing specific services to the researchers seeking ways for supplying their services needs through specialized corporations is absent currently, albeit an efficient example exists within the Nanotechnology Network of Iran. Unspecialized function of the incubators and science and technology parks in the defined fields render them non professional and inefficient, for example Tehran University of Medical Sciences has established its incubator (pharmaceutical product development center) as a specialized incubator. Think tanks, commercialization consultation centers and idea evaluation centers are absent. As mentioned earlier, the commercialization and entrepreneurial activity in the biopharmaceutical sector depends upon the national economic growth. So, further growth in biopharmaceutical sector is subject to macroeconomic policies. An industrialist said:

"In comparison, most countries experiencing considerable biotechnology growth, also experience high economic growth rates."

#### Solutions

Commercialization and entrepreneurship advocacy can contribute to more extended efforts by the researchers and students through to the goal. One policy maker said:

"We have not learned entrepreneurship in the schools and feel stranger with that concept."

Establishing offices dedicated to entrepreneurial facilitations in universities would put a breakthrough to the present situation. Management practices revision as mentioned in the first part is recommended by the experts. Active pathways for informing the researchers about the policies and regulations in concordance with introducing further transparency in the management are suggested.

### Industrial manufacturing capacity

#### Challenges

The pharmaceutical industry is mainly possess by the government and its related bodies, most companies are outdated ones and should undergo renewal and reconstruction to cope with continuously tightening current good manufacturing practice (cGMP) requirements and can hardly absorb new technologies that may be ascribed partly to management practices applied and production capabilities. However the few biopharmaceutical companies are private. Not sufficient are the experienced, well educated and innovative middle and top level managers in the industry. Knowledge and technology management, organizational excellence models, innovation friendly atmosphere are not frequent in the industry but some movements have formed recently. An academic staff with industrial experience said:

"Managers in the biopharmaceutical industry are, most, not science and technology oriented enough to attract innovation."

Strategic alliances and clusters are new to the industry. However uncompetitive domestic market hinders quality improvement.

#### Solutions

Biopharmaceutical industry as the direct interface between biopharmaceutical innovation and the market requires attention. Privatization of the industry and the total economy is a necessity. An industrialist said:

"Till the economy is ruled by the government no major outbreak in the pharmaceutical and bio industry is probable."

Biopharmaceutical industry human resources should also be well replaced and trained.

### Promoting values and the public culture

#### Challenges

Cultural factors are important items that determine the background and context for entrepreneurial activity in the society, as emphasized by most academic staff and policy makers.

One academic staff stressed that:

"Nowadays most researchers prefer rather short term and fast resulting fields. Long term investment has not yet been prevalent and most investors prefer fields with immediate profitability, which can hinder the vital R&D time and long term investment."

Some research policy makers believed that:

"Policy makers, on the other hand, should follow and foresee the trends in the industry and research field to prepare for the situations coming forth as well as the researchers and industrial activists."

Being bound to team work culture was shown to be a key issue that could render the current research capacity more efficient and fruitful. This issue is not so prevalent in the universities and research institutes. Most interviewees shed light on the fact that team work is a must:

"We should oblige ourselves to team work, an ignored necessity."

Values like perseverance, lack of egoism, not exaggerating the achievements, team work and international cooperation are necessary for the whole system; as most interviewees mentioned. Industrial activists, especially, pointed at some basic cultural determinants like consuming the national products and self esteem in the provision of the domestic market needs and meanwhile focusing efforts towards export of science based and high technology products that should be fixed in the whole society of the country. Another considerable aspect, mentioned iteratively by the academic staff, is the promotion of innovation and the need for change in the student and researchers attitudes. Regarding the fundamental impact of seeking innovation on the commercialization and applicability of the researches being conducted, one can comprehend the influence of people looking for new products and process on the overall economic growth in the biopharmaceutical sector. One of the policy makers stated that:

"We, naturally, try to copy and imitate others efforts; not looking for being different."

Policy makers and industrial activists stated that risk taking and continuous improvement among the managers is drastically not evident, a matter that negatively affects the organizational development especially in the high technology fields. Evidence based policy and decision making, as proposed by most interviewees, is capable of lessening a great deal of issues.

#### Solutions

Providence and long term thinking in the research and development should be incorporated. Perseverance in the applied fields of research helps the commercialization process. “Management by values (MBV) principles” in the organizations such as universities and small and medium-sized enterprises (SMEs) can also promote the entrepreneurial motions.

Cultural modifications require the involvement of top level bodies responsible for the public culture to act properly. Being free of any obligations for the selection of the study discipline is somehow approached as the basic element to allow the researchers function properly. One policy maker said:

"Most students have to achieve ambitions and wishes of their parents."

Innovative ideation can promote novel and modern research areas and technologies for the biopharmaceutical industry.

### Extending the relations and communications

#### Challenges

Miscellaneous efforts in the universities and contemporary efforts in the industry are being done but not cooperating to synergize the expenditure and time. Approach toward scientific and technological cooperation among the activists from both sides must be modified and the false self sufficiency be exchanged by the interactionism. Brokering is neglected in the Iranian biopharmaceutical industry as well as other industries. One of the industrialists said:

"We approach the brokers as negative characters while they can help us."

Communication with the pioneering sources including outstanding universities, international biopharmaceutical companies and consultancy incorporations are not expanded as much as needed. Most experts but especially industrialists laid stress on the scientific communications to extrapolate biotechnology commercialization advocacy among the researchers. Relations between the authorities and policy making and regulatory bodies were perceived as cornerstone aspect for the biopharmaceutical commercialization that functions unsatisfactorily. Adopting consistent and stable policies within different authorities specifically on the manufacturing policies is an important challenge, although firm centralization in legislation and policy making is presently applied. It was regarded as a crucial point that international relations of the country drastically influenced the whole economy and as a sector, the biopharmaceutical industry. A science and technology policy maker cited:

“We cannot restrict ourselves in the national borders; since, today, the product and raw material market is an international one.”

Scientific communications with developed countries, regional and international organizations relating to biotechnology, entering commercial and industrial treaties with pioneers, extending the market beyond the national and local boundaries, among others, are all heavily overshadowed by the extent to which the international relations may protrude.

#### Solutions

Well managed university and biopharmaceutical industry relation was mentioned as an obligation. Interaction and partnership in research among scientists from different fields relating to the biopharmaceutical industry was deemed to be helpful. Some academic staff stressed that:

“There are so many research problems that can be solved only through scientific cooperation.”

As the literature on knowledge translation denotes, active characterization and probing into the real knowledge and technology needs can make remarkable assistance to the commercialization process, a role which may be played satisfactorily by the brokers. Training in fields like commercial management and international marketing can also notably contribute to further extend the relations. Technology transfer might be perceived as a suitable choice for making up the delay in the knowledge and technology creation by the research institutes. One policy maker stated that:

“We can compensate our lag in biotechnology by transferring the critical technologies.”

## Discussion

Biotechnology as a fast growing source of new technologies and innovative, safer, targeted and curative medicines for the pharmaceutical industries has obtained attention [1]. Biopharmaceuticals accounts for an increasing part of the expenditure on the pharmaceuticals. The specific feature of biotechnology, in general, in being based largely on academic and university researches renders the commercialization of research findings substantial importance. There are a satisfactory number of research institutes in the biopharmaceutical field in Iran, but the number of biopharmaceutical companies has been limited to few ones. One possible explanation for the perceived gap between research and production might be the malfunction of the commercialization practice [8,16]. All interviewees perceived university research finding commercialization as necessary as the researches conducted in the biopharmaceutical field [17,18]. They expressed that expending research budget on researches that were not to be applicable and help the country solve any problem, is of no significance, a situation referred to as valley of death for research. There was consensus on these facts: there are no adequate research budgets and grants while private sector is so weak, purposeful and applied researches are rare, commercialization underpinned no structured and organized system, communications in/outside the sector and international ones dose not fulfill the requisites, entrepreneurial facilitations and knowledge is shortcoming and cultural basis is lacking [19]. Amendment and revision in policies, regulation and organizational management, as the most critical issue expressed, in relating grounds such as research, communications, entrepreneurial and commercialization procedures must be considered [20]. Moving towards free market and economic privatization was mentioned, requiring the lessened involvement of the government in medicine market. Regarding the complex and multi faceted nature of the commercialization process and to gather the more of the stakeholder’s approaches toward the topic for provision of a policy making aid and not testing hypotheses, a qualitative method was chosen to discover and develop more detailed body of knowledge on the issue [21,22]. Sampling was based on the stakeholders engaged in a purposeful way, while, perhaps naturally, the formal positions held influenced their responding pattern and attitudes, particularly those serving as policy makers and top level managers of the governmental authorities, as qualitative methodology presupposes. However, very few studies have specifically investigated the biopharmaceutical commercialization process in developing countries [23]. Various background types like industrialists, policy making and researchers focused on different aspect of the commercialization process. Technical biotechnology issues, pricing policy and market competition regards were emphasized by the industrialists while policy makers mainly highlighted the social and public benefits for higher quality and moderate price and researchers underlined grants and entrepreneurial aspects [24]. Although some experts who were involved in more than one background like research and industry entered the study, a fact that eased the inter profession interpretation. However, each background potentiated some approaches to some extent, especially in the industrialists. We tried to include the most comprehensive informant society of the research and industry and policy making sides, however, some conflicts of interest and bias were noted and cared. Governmental interventions on the medicine market control, pricing and policy making raised some disagreements, which the impact of the interviewee position was evident on. Different data gathering methodologies fairly increased the data accuracy and trustworthiness by triangulation of data sources [25] as well as the construct validity of the present study [26]. Ireland et al. [27] have studies different new biotechnology firms and developed a conceptual model to delineate how they had organized business and science issues. They have concluded that the successful firms could have balanced their scientific and business issues. Terziovski et al. [28] have investigated management practices and strategies that are critical for successful commercialization in the biotechnology industry. They have also specifies the challenges faced by the biotechnology industry in human resources, financial issues, entrepreneurial and business skills and supply chain linkages, however they have focused on the industry rather than the university. Markman et al. [29] studied the factors that could affect the speed of university technology to the market. They found some factors that influenced technology commercialization in the university side of the commercialization process. They shed light on the capabilities and resources available for the university technology transfer offices (UTTOs) and their impact on the commercialization speed. Fontes [12] has studied the role that biotechnology spin-offs could play in the commercialization of the knowledge produced by the research organizations (ROs). It was shown that these spin-offs can be regarded as alternatives for technology transfer offices. The present study tries to provide a comprehensive approach into the commercialization of biopharmaceutical knowledge. Major barriers and challenges in the successful commercialization process in Iran were identified and the most influential factors for overcoming the challenges were introduced. The results imply that national innovation system can be respected as a suitable model to go for investigating the barriers and providing facilitators for commercialization practice [30-33].

## Conclusion

The present study tries to discover the major factors that influence the commercialization of the biopharmaceutical knowledge and propose processes for moving research to marketplace. As the nature of qualitative study denotes, results derived from one situation could hardly be extrapolated to other ones, but some general issues can be highlighted. Management practices, regulations particularly on the intellectual property right and policy making affects the entire commercialization process even the research as the antecedent. Financial support for the researches is a factor that can facilitate commercialization [18]. Globally, few university researches after long time may be commercialized; therefore quantity and quality of the researches and their applicability may be respected. Human resource management plays an important role in the biopharmaceutical industry, as a high tech field especially training the HR. Entrepreneurial facilitations can encourage the researchers for commercialization [34]. Cultural factors like trust and team work influence whole the process [35]. University-industry and international relations can affect the applicability of the researches and knowledge transfer, while noticing the disadvantages of university-industry relation [36]. Such a study that goes through this issue for the first time can open the way for further studies that specifically investigates each factor. Nearly all the interviewees stated that the commercialization process has been facilitated deeply during recent years, but there are others to do to achieve what is desired. Iran as a developing country can be regarded as a pattern for other developing countries while specific local situations being kept in mind. We can conclude here that the further potentiation of the national innovation system can vitally facilitate the commercialization of biopharmaceutical knowledge.

## Consent

Written consent was obtained from the interviewees for tape recording the interviews and the publication of this report.

## Abbreviations

NIS: National Innovation System; MOHME: Ministry of Health and Medical Education; HR: Human Resource; INIC: Iranian Nanotechnology Initiative Council; R&D: Research and Development; cGMP: Current Good Manufacturing Practice; MBV: Management by Values; SMEs: Small and Medium-sized Enterprises; UTTOs: University Technology Transfer Offices; ROs: Research Organizations.

## Competing interests

The authors declare that they have no competing interests.

## Authors’ contributions

NNK conceived and implemented the strategy, performed the interviews and data analysis and drafted the paper, RM conceived the strategy of study and supervised the project, AK revised the strategy and supervised the project, AR gave consultation on study design and qualitative methodology and edited the draft, MTY gave consultation on biopharmaceutical industry, SN gave consultation on designing the study and qualitative methodology, SN gave consultation on the study implementation and edited the draft. All authors read and approved the final manuscript.

## References

[B1] Hulse JH Biotechnologies: past history, present state and future prospects Trends Food Sci Tech 2004 15 3 18 10.1016/S0924-2244(03)00157-2

[B2] Kayser O Warzecha H Pharmaceutical Biotechnology: Drug Discovery and Clinical Applications 2012 John Wiley-VCH verlag: Weinheim

[B3] Friedl KE Overcoming the "valley of death": mouse models to accelerate translational research Diabetes Technol Ther 2006 8 3 413 414 10.1089/dia.2006.8.413 16800763

[B4] Powers JB Commercializing academic research: Resource effects on performance of university technology transfer J Higher Educ 2003 74 1 26 50 10.1353/jhe.2003.0005

[B5] Kebriaeezadeh A Nassiri-Koopaei N Abdollahias A Nikfar S Nafiseh Mohamadi Trend analysis of the pharmaceutical market in Iran; 1997–2010; policy implications for developing countries DARU 2013 21 1 52 10.1186/2008-2231-21-52 23805853 PMC3718624

[B6] Nikfar S Kebriaeezadeh A Dinarvand R Abdollahi M Sahraian MA Henry D Akbari Sari A Cost-effectiveness of different interferon beta products for relapsing-remitting and secondary progressive multiple sclerosis: decision analysis based on long-term clinical data and switchable treatments DARU 2013 21 1 50 10.1186/2008-2231-21-50 23800250 PMC3698128

[B7] Nikfar S Kebriaeezadeh A Majdzadeh R Abdollahi M Monitoring of National Drug Policy (NDP) and its standardized indicators; conformity to decisions of the national drug selecting committee in Iran BMC Int Health Hum Rights 2005 5 1 5 10.1186/1472-698X-5-5 15885139 PMC1145184

[B8] Lichtenthaler U Lichtenthaler E Frishammar J Technology commercialization intelligence: organizational antecedents and performance consequences Technol Forecast Soc Change 2009 76 301 315 10.1016/j.techfore.2008.07.002

[B9] Hashemi Meshkini A Kebriaeezadeh A Dinarvand R Nikfar S Habibzadeh MG Vazirian I Assessment of the vaccine industry in Iran in context of accession to WTO: a survey study DARU 2012 20 19 10.1186/2008-2231-20-19 23351841 PMC3555710

[B10] Szelényi K Goldberg RA Commercial funding in academe: examining the correlates of faculty's use of industrial and business funding for academic work J Higher Edu 2011 82 6 775 799 10.1353/jhe.2011.0040

[B11] Clarysse B Tartari V Salter A The impact of entrepreneurial capacity, experience and organizational support on academic entrepreneurship Res Policy 2011 40 8 1084 1093 10.1016/j.respol.2011.05.010

[B12] Fontes M The process of transformation of scientific and technological knowledge into economic value conducted by biotechnology spin-offs Technovation 2005 25 339 347 10.1016/j.technovation.2003.08.004

[B13] Davari M Walley T Haycox A Pharmaceutical policy and market in Iran: Past experiences and future challenges J Pharm Health Serv Res 2011 2 1 47 52 10.1111/j.1759-8893.2011.00042.x

[B14] Majdzadeh R Sadighi J Nejat S Shahidzade Mahani A Gholami J Knowledge Translation for Research Utilization: design of a Knowledge Translation Model at Tehran University of Medical Sciences J Contin Educ Health Prof 2008 28 4 270 277 10.1002/chp.193 19058259

[B15] Patton MQ Qualitative research and evaluation methods 2001 Thousand Oaks, CA: Sage Publications

[B16] Goldfarb B Henrekson M Bottom-up versus top-down policies towards the commercialization of university intellectual property Res Policy 2003 32 4 639 658 10.1016/S0048-7333(02)00034-3

[B17] Onyeka CJ Biotechnology commercialisation in universities of developing countries: a review of the University of Ibadan Nigeria. J Commer Biotechnol 2011 17 4 293 300 10.1057/jcb.2011.28

[B18] Olivieri NF Patients' health or company profits? The commercialisation of academic research Sci Eng Ethics 2003 9 1 29 41 10.1007/s11948-003-0017-x 12645227

[B19] Meyers AD Pruthi S Academic entrepreneurship, entrepreneurial universities and biotechnology J Commer Biotechnol 2011 17 4 349 357 10.1057/jcb.2011.22

[B20] Gilsing VA van Burg E Romme AGL Policy principles for the creation and success of corporate and academic spin-offs Technovation 2010 30 1 12 23 10.1016/j.technovation.2009.07.004

[B21] Hall ZW Scott C University-industry partnership Science 2001 291 5504 553 10.1126/science.1058411 11229387

[B22] Kharabaf S Abdollahi M Science growth in Iran over the past 35 years J Res Med Sci 2012 17 3 1 5 23267381 PMC3527047

[B23] De Luca LM Verona G Vicari S Market orientation and R and D effectiveness in high-technology firms: an empirical investigation in the biotechnology industry J Prod Innovat Manag 2010 27 3 299 320 10.1111/j.1540-5885.2010.00718.x

[B24] Bureth A Pénin J Wolff S Start-up creation in biotechnology: lessons from the case of four new ventures in the upper rhine biovalley IJIM 2010 14 2 253 283

[B25] Tashakkori A Teddlie C Handbook of mixed methods in social and behavioral research 2002 Thousand Oaks, CA: Sage Publications

[B26] Yin RK Case Study Research: Design and Methods 2002 Thousand Oaks, CA: Sage Publications

[B27] Ireland DC Hine D Harmonizing science and business agendas for growth in new biotechnology firms: case comparisons from five countries Technovation 2007 27 676 692 10.1016/j.technovation.2007.05.016

[B28] Terziovski M Morgan JP Management practices and strategies to accelerate the innovation cycle in the biotechnology industry Technovation 2006 26 5–6 545 552

[B29] Markman GD Gianiodis PT Phan PH Balkin DB Innovation speed: transferring university technology to market Res Policy 2005 34 7 1058 1075 10.1016/j.respol.2005.05.007

[B30] Larijani B Majdzadeh R Delavari AR Rajabi F Khatibzadeh S Esmailzadeh H Lankarani KB Iran's health innovation and science development plan by 2025 Iran J Public Health 2009 38 SUPPL. 1 13 16

[B31] Bérard C Delerue H A cross-cultural analysis of intellectual asset protection in SMEs: the effect of environmental scanning J Small Bus Enterprise Dev 2010 17 2 167 183 10.1108/14626001011041193

[B32] Eisenberg RS Patent costs and unlicensed use of patented inventions Univ Chic Law Rev 2011 78 1 53 69

[B33] Bagheri SK Moradpour HA Rezapour M The Iranian patent reform World Pat Inf 2009 31 1 32 35 10.1016/j.wpi.2008.04.008

[B34] Jain S George G Maltarich M Academics or entrepreneurs? Investigating role identity modification of university scientists involved in commercialization activity Res Policy 2009 38 922 935 10.1016/j.respol.2009.02.007

[B35] Fiedler M Welpe IM Commercialisation of technology innovations: an empirical study on the influence of clusters and innovation networks Int J Technol Manag 2011 54 4 410 437 10.1504/IJTM.2011.041582

[B36] Kumar MN Ethical conflicts in commercialization of university research in the post-bayh-dole era Ethics Behav 2010 20 5 324 351 10.1080/10508422.2010.491759

